# The role of long-acting antipsychotics in illness relapse: an observational study

**DOI:** 10.1192/j.eurpsy.2024.805

**Published:** 2024-08-27

**Authors:** I. Berardelli, I. Mancinelli, E. Rogante, D. Erbuto, M. A. Trocchia, L. Longhini, L. Rapisarda, A. Bruzzese, S. Sarubbi, M. Pompili

**Affiliations:** ^1^Neurosciences, Mental Health and Sensory Organs, Sant’ Andrea Hospital, Sapienza University of Rome; ^2^Neurosciences, Mental Health and Sensory Organs, Sant’ Andrea Hospital; ^3^Human Neuroscience, Sapienza University of Rome; ^4^Psychiatry Residency Training Program, Sant’ Andrea Hospital, Sapienza University of Rome, Rome, Italy

## Abstract

**Introduction:**

In patients affected by Schizophrenia and Bipolar Disorder disorders the use of antipsychotic drugs is essential in preventing the exacerbation of symptoms. The use of long-acting injectable (LAI) antipsychotics is considered an important treatment option. The aim of this study was to evaluate the incidence and predictors of relapse during antipsychotic treatment with LAIs in a sample of psychiatric outpatients up to a year after the start of long-acting therapy.

**Objectives:**

The study included 103 adult patients admitted to the psychiatric unit of Sant’Andrea University Hospital in Rome.

**Methods:**

We evaluated duration of untreated illness, previous treatments, substance abuse, suicidal status, LAI dose, and use of other medicines for association with new episodes of illness or of symptomatic worsening as well as hospitalization, using bivariate and multivariate analyses.

**Results:**

Seventy-three patients were diagnosed with schizophrenia spectrum and 30 with bipolar disorders. Age at study entry averaged 36.7 years (SD= 11.55). 40.8% of patients were women. The mean age at onset were 23.11 (SD= 7.0). All the other information were reported in Table 1. On 103 patients undergoing with LAI treatment for a year only 9 (8.7%) patients had a relapse during the study period. The two groups differed according to the presence of hospitalization during the 12 months before the LAI treatment (p = .022), in particular patients with relapse were more hospitalized than patients with no relapse (62.5% vs. 21.7%). Moreover, group with relapse were more at risk of suicide during the 12 months before the LAI treatment than the other group, for both suicidal ideation (11.1% vs. 4.3%; p= .015) and attempt (25.0% vs. 3.2%; p= .049). Finally, the two groups differed according to the side effects reported during the year of LAI treatment (χ² =38.48; p< .001). Specifically, patients’ group with relapse reported more side effects caused by parkinsonism (25.0% vs. 1.1%) and tremor (25.0% vs. 0%). No differences were found for the other variables (See table 1).

**Image:**

**Figure:**
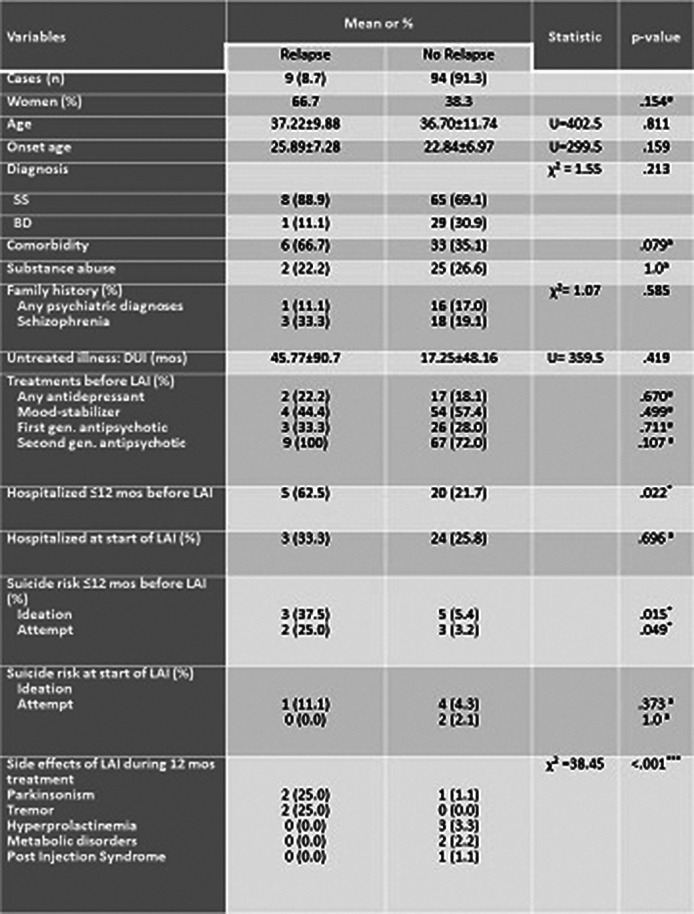

**Conclusions:**

In conclusion, our observations confirm the importance of LAI therapy in real word. However, our results indicate that these drugs might not prevent subsequent exacerbations for a proportion of individuals whose illness is stabilised on continuous antipsychotic treatment. Extra pyramidal symptoms in particular might have pathophysiological implications for relapse.

**Disclosure of Interest:**

None Declared

